# Structure and membership of gut microbial communities in multiple fish cryptic species under potential migratory effects

**DOI:** 10.1038/s41598-020-64570-8

**Published:** 2020-05-05

**Authors:** My Hanh Le, Daryi Wang

**Affiliations:** 1 Biodiversity Research Center, Academia Sinica, Taipei, Taiwan; 2 Department of Life Science, National Taiwan Normal University, Taipei, Taiwan; 3 Biodiversity Program, Taiwan International Graduate Program, Academia Sinica and National Taiwan Normal University, Taipei, Taiwan; 4 Institute of Ecology and Biological Resources, Vietnam Academy of Science and Technology, Hanoi, Viet Nam

**Keywords:** Microbial ecology, Microbial communities

## Abstract

The animal gut microbiota evolves quickly towards a complex community and plays crucial roles in its host’s health and development. Factors such as host genetics and environmental changes are regarded as important for controlling the dynamics of animal gut microbiota. Migratory animals are an important group for studying how these factors influence gut microbiota because they experience strong environmental perturbations during migration. The commercially important grey mullet, *Mugil cephalus*, is a cosmopolitan species complex that display reproductive migration behaviour. There are three cryptic species of *M. cephalus* fish distributed across the Northwest Pacific, and their spawning sites overlap in the Taiwan Strait. This extraordinary natural occurrence makes the grey mullet an ideal model organism for exploring the nature of wild animal-gut microbiota relationships and interactions. This study investigates the diversity and structure of the gut microbial community in three cryptic *M. cephalus* species using 16S rRNA amplicon sequencing. Gut microbial compositions from adult and juvenile fish samples were analysed. Our results indicate that gut microbial communities within the grey mullet share a core microbiome dominated by *Proteobacteria, Firmicutes* and *Actinobacteria*. However, the structures of gut microbial communities were more distinct between adult mullet groups than they were between juvenile ones. Intriguingly, we found that adult fish that migrate to different geographical tracts harbour gut microbiota similar to historical records of seawater microflora, along their respective migration routes. This observation provides new insights into the interaction between aquatic animal gut microbial communities and the environments along their hosts’ migratory routes, and thus warrants future study.

## Introduction

The animal gastrointestinal tract harbours vast and dynamic populations of microbes known as intestinal microbiota. Intestinal microbiota plays an important role in host development and immunity; its structure and some of its special functions can be shaped by multiple endogenous factors in the host and exogenous influences from the environment^1,2^. It has been suggested that the symbiotic microbial community is largely determined by its host’s genetic background, and gut microbial compositions between hosts of the same species or family are more similar than between those of different host species or families^3^. Growing evidence suggests that the hosts’ genetics influence their gut microbial compositions, which can maintain the stability of the gut microbial community membership throughout the life of an organism, or change as a result of strong perturbations^4^. Moreover, this supports the hypothesis that gut microbial composition plasticity, especially in special external environmental conditions, facilitates host acclimation and adaptation^5,6^. Yet, even though we have a considerable understanding of the interaction between animals and their gut microbiota, the extent to which the environment and host genetics contribute to shaping the microbiota across the host’s life remains unaccounted for.

Animal migration occurs seasonally—mainly due to food availability, or for breeding purposes—and has been observed in various groups including birds, mammals, reptiles, amphibians, insects, and fish^7^. Accordingly, migration is considered an ideal event for investigating not only the long-term influence of environmental conditions and food sources in an organism’s original habitat, but also the temporal impacts of external factors and available food on the intestinal microbial communities of wild animals^8,9^. The migration process requires migratory animals to face physiological challenges, during which time the host body might need to store huge amounts of energy to remain active for an extended period of time^10^. The interaction between migration and the structure of gut microbial compositions in migratory birds was investigated previously. Lewis *et al*.^8^ documented that three different migratory bird species that shared the same stopover field had similar gut microbial communities, which suggests that temporal environmental and food conditions have a strong impact on the structure of gut microbiota in some migratory bird species during stopovers. However, a recent study provided evidence that animal gut microbial communities offer resistance during their hosts’ migration; the study came to this conclusion after finding a high similarity between the gut microbiota of stint flocks migrating thousands of kilometres and resident birds that had inhabited the same field for a full year^9^. These observations suggest that migratory bird gut microbiota are shaped by complex interactions among multiple factors.

Nevertheless, many migratory fish species are different from migratory birds and mammals in that they accumulate energy for months before migrating and rarely consume food during long-distance migration. This is mainly because their feeding behaviour—e.g. foraging for and digesting food—limits their ability to migrate^11^. In addition, migratory fishes not only undergo concurrent changes in host physiology, geography, and diet, but also come in direct contact with a high diversity of microorganisms in water environments along their migration route. However, there have been no studies on how migration influences the structure of gut microbiota in migratory fish or host-microbe interactions. Previous studies found that both the core microbiota composition in the Atlantic salmon digestive tract and shifts/changes in its structure are correlated with the host’s transfer from freshwater to seawater^12,13^. Although these laboratory-based studies on wild hosts may help unravel how rapid environmental change leads to drastic changes in host gut microbiota, such studies may not truly reflect gut microbiotas’ responses to environmental disturbances under natural conditions.

**Figure 1:**
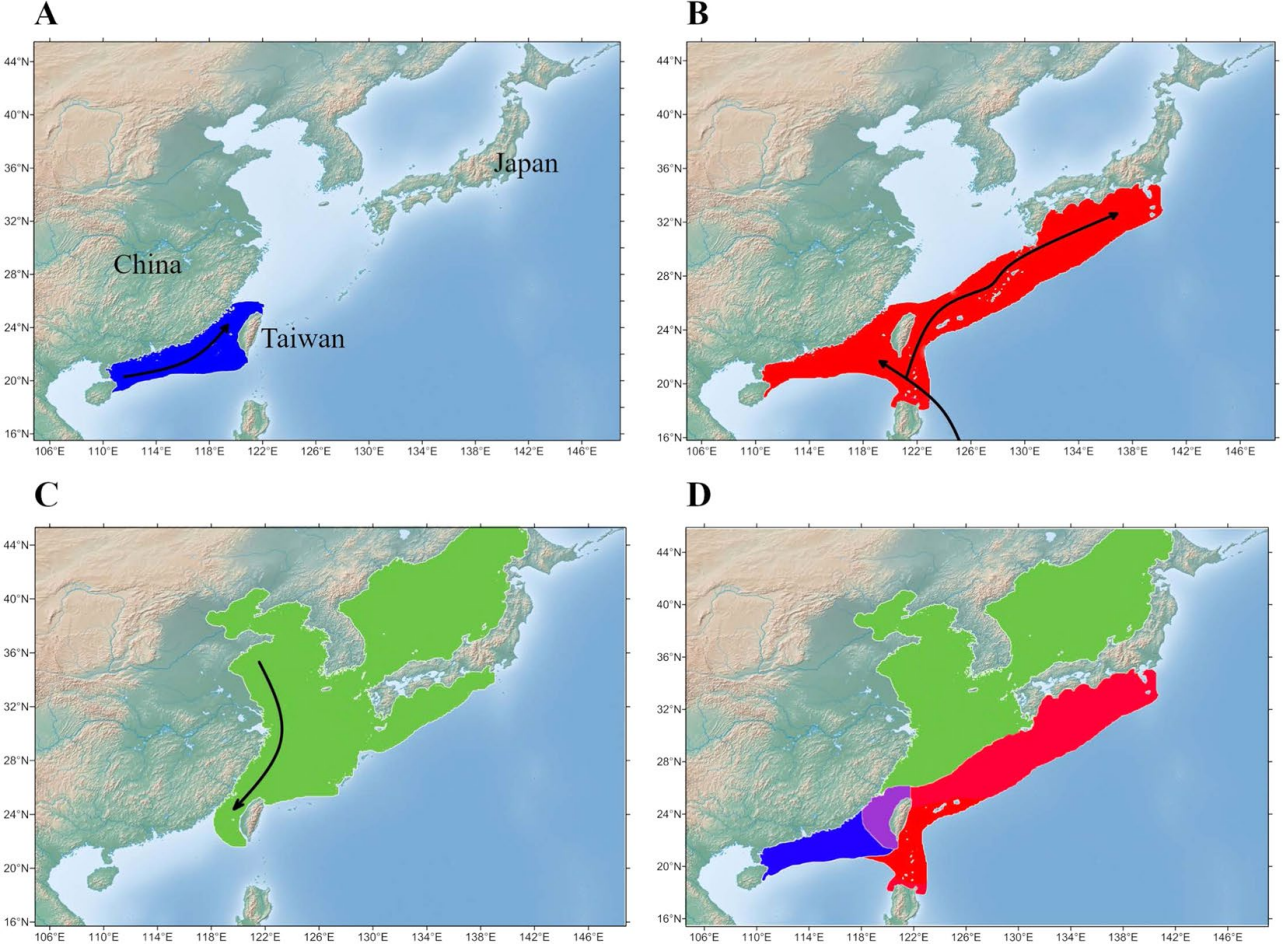
The proposed distribution and migration routes in the Northwest Pacific (NWP) of three cryptic species of *Mugil cephalus* to their spawning area in the Taiwan Strait. (**A**) NWP1’s distribution (green) and migration route from the coast of China. (**B**) NWP2’s distribution (red) and migration route following the Kuroshio Current. (**C**) NWP3’s distribution (blue) and migration direction from the SCS. (**D**) The distribution map of three cryptic species; purple indicates the spawning area shared among the three *M. cephalus* cryptic species in the Taiwan Strait. The maps were adapted from Shen *et al*., 2011^15^ and created in GenGIS software^58^.

The grey mullet (*Mugil cephalus* L.) is an important species for commercial fishing and aquaculture; it is widely distributed and considered to have a catadromous life history. Interestingly, previous studies have suggested that *M. cephalus* is a species complex (super species) with at least 14 cryptic species around the world^14^. Of these, three cryptic grey mullet species spawn in the Taiwan Strait (spawning season: approximately November to January). The migratory tracts of these three cryptic species (denoted NWP1, NWP2 and NWP3) are believed to be in the Northwest Pacific (Fig. 1)^15^. In brief, Shen and his co-workers analysed over 700 grey mullet samples from 12 locations in the East and South China Seas (SCS) using mtDNA Cytochrome Oxidase I (COI) sequences and ten microsatellite loci, and indicated that cryptic species NWP1 migrates to the Taiwan Strait from estuarine environments and adjacent areas in the East China Sea (ECS), whereas NWP2 and NWP3 migrate following the Kuroshio Currents from the SCS in the middle of the winter. Consistent with other migratory fish species, grey mullet adults are usually observed to have empty stomachs, which suggests that the grey mullet also does not consume food during migration. Young grey mullets of all three cryptic species develop in the waters around Taiwan until they reach the appropriate size to migrate to their ideal nursing areas. The grey mullet *M. cephalus* has three known cryptic species that use the Taiwan Strait for spawning and have fascinating migratory histories, making them ideal model species for investigating whether the divergence in gut microbiota is triggered by the influence of host genetic background and migration.

Here, we studied the gut microbiota of both juveniles and adults of three cryptic species in the *Mugil cephalus* species complex that spawned in Taiwanese waters to investigate the importance of host genetics and migration history. We hypothesized that migration impacts the gut microbiota. We predicted that adult fishes that migrated from different areas in the Northwest Pacific into Taiwanese waters would have distinct gut microbiota but their juveniles that spawned in Taiwanese waters would share similar gut microbiota. Alternatively, if host genetics were an important factor, then all fish from the species complex—including juveniles and adults—would share the same core microbiota that are resistant to perturbations from migration histories. In addition, we compared the gut microbial community of the grey mullet with historical records of the microbiomes of seawater along their suggested geographical migration tracts in the Northwest Pacific to explore the potential impact of seawater on the gut microbiota of migratory fish.

## Results

### General features of collected samples and characteristics of sequenced data

We analysed a total 16 adult samples (NWP1 n = 8; NWP2 n = 4; NWP3 n = 4) and 12 juvenile samples (NWP1 n = 6; NWP2 n = 6) from three cryptic species (NWP1, NWP2, and NWP3) in the grey mullet *M. cephalus* species complex in the Taiwan Strait during their spawning season. Fish samples were identified as specific cryptic species based on methods from previous studies^15,16^ (see Materials and Methods). The diversity and structure of the gut microbial composition were determined by performing deep sequencing of bacterial 16S rRNA genes from all fish gut samples. After quality filtering and removing chimeras and single reads, a total of 3,704,851 high-quality reads were obtained from 16 adult (hereafter AD) and 12 juvenile (JV) grey mullet samples. The number of reads ranged from 75,613 to 200,144 reads of 16S rRNA amplicons in each sample (Supplementary Table 1), resulting in the identification of 1,160 operational taxonomic units (OTUs).

### Evaluation of the microbial complexity in the *M. cephalus* species complex

**Figure 2:**
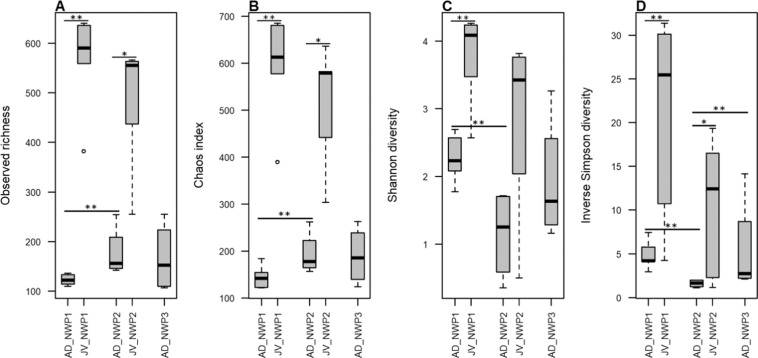
Alpha diversity in the grey mullet gut microbiota. (**A**) Observed richness, (**B**) Chaos diversity index, (**C**) Shannon diversity, and (**D**) inverse Simpson diversity indexes were calculated following rarefaction-based normalization of the OTU table. Significant *p* values (*p < 0.05; **p < 0.01) were obtained using a permutation T-test. X axis shows the group IDs; JV is juvenile, AD is adult, NWP1 is cryptic species 1, NWP2 is cryptic species 2, and NWP3 is cryptic species 3.

In this study, 750,613 reads were rarefied in all samples. Rarefaction curves approached the saturation plateau and adequately represented the gut microbial community diversity in all samples (Supplementary Fig. 1). Although there was no difference between the results of the analyses using rarefied and unrarefied data, it is believed that rarefied data can ignore the presence of rare species, which leads to false positives^17^. Therefore, we prioritized the results from the unrarefied data. At the species level, wild AD grey mullets had an average of 123 ± 9, 177 ± 39, and 166 ± 57 OTUs in NWP1, NWP2, and NWP3, respectively. Meanwhile, we detected 566 ± 64 OTUs in NWP1 and 489 ± 95 OTUs in NWP2 juveniles. Inter-specific variation among individuals in sampled species were examined using a permutational test for homogeneity of multivariate dispersions. Although the box plot showed higher inter-variation between individual samples of NWP3 juvenile adult (Supplementary Fig. 2), the *p* value was not significant (*p* = 0.292). The alpha diversity—including observed richness (sob), Chao diversity, Shannon index (H’) and inverse Simpson diversity (S) indices—were calculated. In addition, permutation T-test was conducted to test whether there was any difference in alpha diversity among adult groups, among juvenile groups, and between adults and juveniles of the same cryptic species. The results showed that, while there are similarities between the alpha diversities of AD_NWP1 and AD_NWP3 and between JV_NWP1 and JV_NWP2 in both indices, there was significantly more diversity in juvenile samples compared to adults of the same cryptic species (AD_NWP1 – JV_NWP1: *P*_*sob*_ < *0.01, P*_*chao*_ < 0.01, *P*_*H’*_ < 0.01, *P*_*S*_ < 0.01; AD_NWP2 – JV_NWP2: *P*_*sob*_ < *0.05, P*_*chao*_ < 0.05, *P*_*S*_ < 0.05). At the same time, AD_NWP2 had the lowest alpha diversity (except in the Chao estimation) compared to AD_NWP1 in the observed richness and Shannon and Inverse Simpson indices and AD_NWP3 in the Inverse Simpson index (Fig. 2, Supplementary Table 2).

### Gut microbial composition

**Figure 3:**
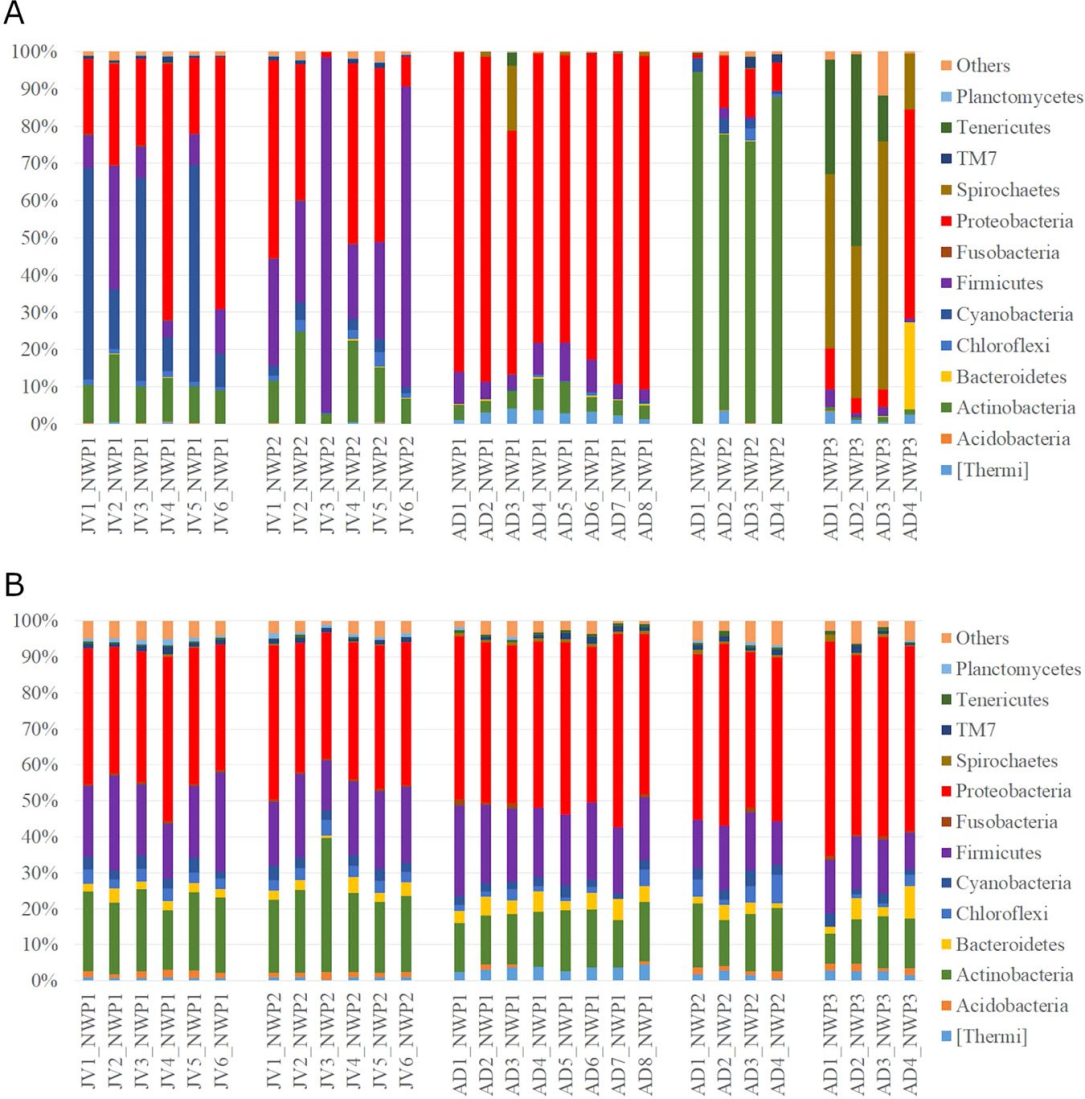
The gut microbial communities in grey mullets at the phylum level. (**A**) The structure of gut microbial compositions (relative abundance of phyla). (**B**) The membership of gut microbial communities (number of taxa present in each phylum category). Others categories include unclassified bacteria and low abundant phyla. X axis shows the sample IDs; JV is juvenile, AD is adult, NWP1 is cryptic species 1, NWP2 is cryptic species 2, and NWP3 is cryptic species 3. There were 12 juvenile samples (NWP1 n = 6; NWP2 n = 6) and 16 adult samples (NWP1 n = 8; NWP2 n = 4; NWP3 n = 4).

The structure of the gastrointestinal microbiota was characterized using the relative abundance of representative sequences from each OTU from each sample to assign a taxonomic classification against the Greengenes database. A total of 45 different cultured and candidate bacterial phyla were detected from all fish gut samples. In adult samples, *M. cephalus* gut microbiota across the phylum were dominated by *Proteobacteria* (65.4–89.5%) in cryptic species 1 (NWP1) while the most abundant phylum of bacteria was *Actinobacteria* (74–94.5%) in cryptic species 2 (NWP2) and *Spirochaetes* (14.8–66.6%) in cryptic species 3 (NWP3). Furthermore, the gut microbiota of juvenile samples NWP1 were dominated by *Proteobacteria* (37.9%), *Cyanobacteria* (33.9%), and *Firmicutes* (12.5%), while the NWP2 juvenile gut microbial community consisted of high abundances of *Firmicutes* (46.4%), *Proteobacteria (*32.3%), and *Actinobacteria* (13.7%) (Fig. 3A).

At the family level, *Moraxellaceae* was the most abundant bacterial group in NWP1 adults (accounting from 43.7–77.7%), while one family in the order *Actinomycetales* dominated 70.2–98.9% of the gut microbial community in NWP2 adults. Samples belonging to cryptic species NWP3 had high abundances of *Brevinemataceae* and *Mycoplasmataceae* bacteria (14.9–92.3%). In juvenile samples, *Streptococcaceae, Clostriaceae*, *Vibrionaceae*, and one unclassified family belonging to order *Stramenopiles* were the most representative of the gut microbial compositions, contributing to 30–94.5% of the total abundance (Supplementary Fig. 3).

In contrast to the relative abundances of gut bacteria, our analysis detected similar proportions of community membership among all grey mullet fish, including adult and juvenile samples. Community membership is calculated as the number of bacteria taxa at the phylum level. Specifically, *Proteobacteria, Firmicutes* and *Actinobacteria* accounted for an average of 44, 19, and 18% of identified OTUs in all fish samples, respectively (Fig. 3B).

**Figure 4:**
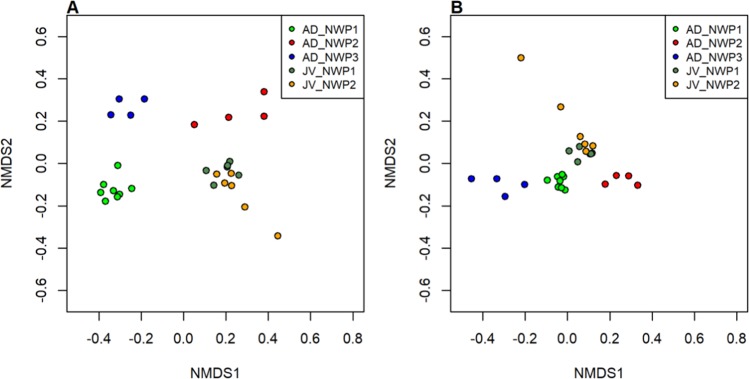
Two-dimensional non-metric multidimensional scaling (NMDS) plots of gut microbial communities. The gut microbiota compositions of 16 adult and 12 juvenile grey mullet samples were used to estimate the similarity between samples based on (**A**). Bray Curtis distance, and (**B**). weighted UniFrac distance. Light green points are gut microbial communities in NWP1 adult samples, red points for communities in NWP2 adults, blue points for communities in NWP3 adults, and dark green and orange dots are juvenile samples of NWP1 and NWP2, respectively. NWP1, NWP2 and NWP3 are three cryptic species of grey mullet *M. cephalus* in the Taiwan Strait.

We next used beta diversity analysis to approximate the variation in gut microbial community structure inside the *M. cephalus* species complex. Non-metric multidimensional scaling (NMDS) plots showed that grey mullet gut microbial compositions were distinctively separate from different cryptic species in adult samples, and were strongly clustered between juvenile fishes based on Bray Curtis and weighted UniFrac distance matrixes (Fig. 4). PERMANOVA analyses also supported our delineated results (Supplementary Table 3). In particular, there were differences in microbial composition among adults of the three cryptic species (*p* < 0.05). The microbial communities in adults and juveniles of the same cryptic species were also significantly different (*p* < 0.001). On the other hand, there were no species-specific differences observed between juvenile samples of NWP1 and NWP2.

### Core gut microbiome of *M. cephalus*

To investigate the existence of a core gut microbiome that is maintained across the *M. cephalus* life history, we focused on 122 genus-level OTUs shared in all the fish groups in this study. Consistent with membership of gut microbiota in grey mullets, most of the core microbes were *Proteobacteria* (56 OTUs, 45.9%), *Firmicutes* (22 OTUs, 18.03%), and *Actinobacteria* (18 OTUs, 14.75%). The fact that the relative abundance of the core gut microbes can be as high as 95.8% in the communities might reflect the importance of bacteria inside the gut microbial community, as well as their potential functions in and benefits to the hosts. Within these 122 OTUs, 35 were present in over 90% of observed samples. Permutation T-test results indicated that many core microbes inside the gut microbial community in AD_NWP1 were significantly different with not only the other adult cryptic species (NWP2 and NWP3; 22 and 13 OTUs, respectively), but also the juveniles of this cryptic species (31 OTUs). OTU499 (assigned to a genus of *Brevinemataceae*) was the only OTU with a notably different abundance among the gut microbiota of all three cryptic species in the adult stage (*P*_*NWP1*–*NWP2*_ < 0.05, *P*_*NWP1*–*NWP3*_ = 0.01, *P*_*NWP2*–*NWP3*_ < 0.05) (Table 1).

**Table 1:** Permutation T-test results of differential abundances of the core microbiome between the gut microbial communities of three cryptic species and their juveniles.

Taxonomy (genus level)	ID	AD1 – AD2	AD1 – AD3	AD2 – AD3	AD1 – JV1	AD2 – JV2	JV1 – JV2
*Actinomycetales_unclassified*	Otu002	**0.006**^**b**^	0.344	**0.026**^**b**^	**0.004**^**a**^	**0.014**^**b**^	0.572
*Bacillus*	Otu017	**0.01**^**a**^	0.176	0.072	0.372	0.302	**0.012** ^**a**^
*Sphingomonadaceae_unclassified*	Otu045	0.368	0.868	0.452	**0.008** ^**b**^	0.222	**0.018** ^**a**^
*Microbacterium*	Otu051	**0.006**^**a**^	**0.012** ^**a**^	0.088	**0.014** ^**b**^	**0.032** ^**b**^	0.47
*Rubrobacter*	Otu092	**0.05**^**a**^	**0.008** ^**a**^	0.934	**0.002** ^**a**^	0.692	0.052
*Psychrobacter*	Otu105	**0.006** ^**a**^	**0.006** ^**a**^	0.634	**0.002** ^**a**^	**0.022** ^**a**^	0.374
*Synechococcus*	Otu209	**0.004** ^**b**^	0.316	**0.03** ^**a**^	**0.006** ^**b**^	**0.024** ^**a**^	**0.014** ^**a**^
*Aeribacillus*	Otu316	**0.006** ^**a**^	**0.006** ^**a**^	0.2	**0.002** ^**a**^	0.738	0.5
*Rhizobiales_unclassified*	Otu003	**0.042** ^**b**^	0.57	0.256	**0.006** ^**b**^	**0.042** ^**b**^	0.216
*Gammaproteobacteria_unclassified*	Otu004	**0.032** ^**b**^	0.06	0.25	0.302	0.07	0.216
*Rhodobacteraceae_unclassified*	Otu005	**0.032** ^**b**^	0.964	0.12	**0.004** ^**b**^	0.604	0.07
*Proteobacteria_unclassified*	Otu006	0.062	0.82	0.712	**0.024** ^**a**^	0.426	**0.02** ^**a**^
*Hyphomicrobium*	Otu030	0.082	0.518	0.078	**0.006** ^**b**^	0.638	0.31
*Deinococcus*	Otu042	**0.016** ^**a**^	0.404	0.146	**0.002** ^**a**^	0.786	0.24
*Comamonadaceae_unclassified*	Otu054	0.06	0.076	0.22	**0.026** ^**b**^	**0.03** ^**b**^	**0.014** ^**a**^
*Sphingomonas*	Otu077	0.276	0.382	0.71	**0.002** ^**a**^	0.274	0.268
*Acinetobacter*	Otu093	0.092	0.638	0.348	**0.004** ^**a**^	0.678	0.28
*Thermus*	Otu337	0.958	0.734	0.96	**0.002** ^**a**^	0.072	0.376
*Brachymonas*	Otu441	**0.008** ^**a**^	**0.01** ^**a**^	0.528	**0.006** ^**a**^	0.54	0.068
*Ralstonia*	Otu496	**0.004** ^**a**^	**0.014** ^**a**^	0.234	**0.002** ^**a**^	0.596	0.978
*Propionibacterium*	Otu505	0.176	**0.01** ^**a**^	0.882	**0.002** ^**a**^	0.568	0.194
*Cupriavidus*	Otu548	**0.022** ^**a**^	0.184	0.142	**0.002** ^**a**^	0.188	0.896
*Bradyrhizobium*	Otu802	**0.012** ^**a**^	**0.006** ^**a**^	0.992	0.472	0.32	0.136
*Methylobacterium*	Otu130	0.43	0.158	0.916	**0.024**^**a**^	0.806	0.07
*02d06*	Otu207	0.554	0.72	0.616	**0.004**^**b**^	**0.006** ^**b**^	0.268
*Anoxybacillus*	Otu498	**0.044** ^**a**^	0.186	0.288	**0.018**^**a**^	0.732	0.642
*Brevinemataceae_unclassified*	Otu499	**0.038** ^**a**^	**0.01** ^**b**^	**0.022** ^**b**^	**0.006**^**a**^	0.218	0.82
*Meiothermus*	Otu554	0.128	0.104	0.97	**0.012**^**a**^	0.764	0.434
*Enhydrobacter*	Otu689	0.766	**0.02**^**a**^	0.952	**0.016**^**a**^	**0.02** ^**b**^	0.472
*Bacteria_unclassified*	Otu001	**0.004**^**b**^	**0.01**^**b**^	0.264	**0.002**^**b**^	0.238	0.578
*Alphaproteobacteria_unclassified*	Otu011	**0.012**^**b**^	0.438	0.086	**0.004**^**b**^	0.746	0.222
*Microbacteriaceae_unclassified*	Otu046	**0.044**^**a**^	**0.012**^**a**^	0.728	0.214	0.386	0.364
*Pseudomonas*	Otu115	**0.004**^**a**^	**0.004**^**a**^	0.14	**0.002**^**a**^	**0.048** ^**b**^	0.7
*Geobacillus*	Otu160	0.354	0.292	0.924	**0.002**^**a**^	0.218	0.22
*Thermoanaerobacterium*	Otu454	**0.032**^**a**^	0.962	**0.05** ^**b**^	**0.03**^**a**^	0.932	0.762
**Total significantly different OTUs**		**22**	**13**	**4**	**31**	**9**	**5**

AD1, NWP1 adult; AD2, NWP2 adult; AD3, NWP3 adult; JV1, NWP1 juvenile; JV2, NWP2 juvenile. NWP1, NWP2 and NWP3 are three cryptic species of the grey mullet M. cephalus in the Taiwan Strait.

^**a**^The former; or ^**b**^the latter component of the comparison was significantly higher.

Statistically significant values are presented in bold.

### Clustering of juvenile gut microbial composition

**Figure 5:**
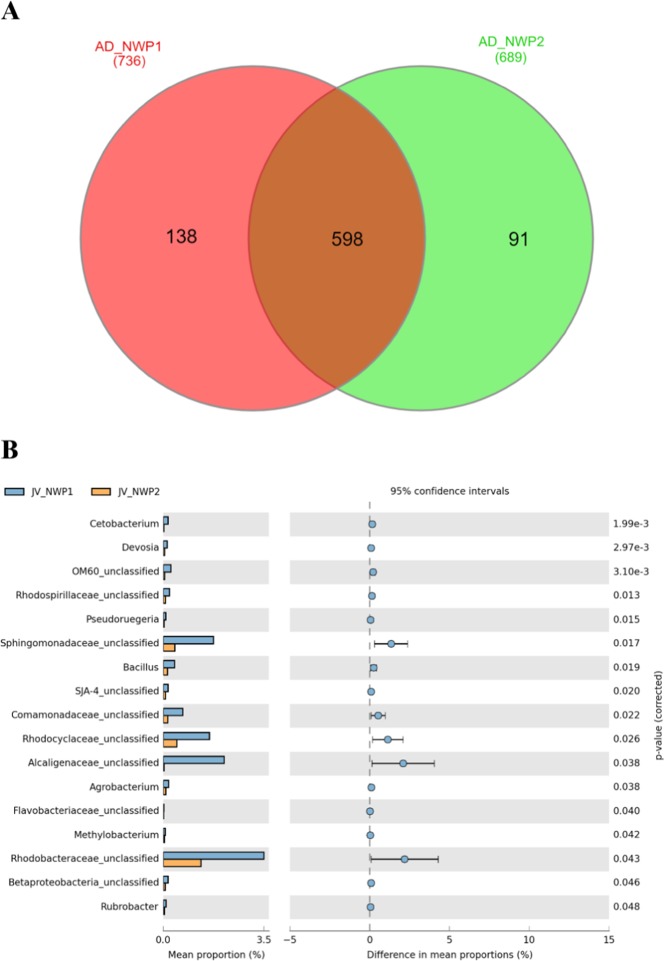
Analysis of the shared OTUs in grey mullet juveniles. (**A**) Venn diagram indicates the unique and shared OTUs between NWP1 juveniles (red orange) and NWP2 juveniles (green). (**B**). Significant different shared OTUs between juveniles of NWP1 (blue) and NWP2 (orange). *p* values were calculated using T-test, and *p* value < 0.05 was considered statistically significant. NWP1 and NWP2 are two cryptic species of grey mullet *M. cephalus* in the Taiwan Strait.

From the 827 OTUs detected at the genus level in the gut microbial communities of 12 juvenile grey mullets (NWP1 n = 6 and NWP2 n = 6), we identified 598 OTUs shared between the two cryptic species, which accounted for 99.75% and 99.87% of the relative abundance in the gut microbial communities of NWP1 and NWP2 juveniles, respectively (Fig. 5A; Supplementary Table 3). High similarities in libraries between juveniles is consistent with the results of the NMDS plots from the Bray-Curtis distant and weighted UniFrac (Fig. 4). In particular, juvenile samples’ gut microbial communities clustered together, and the PERMANOVA test found that they were not significantly different. T-test results showed that only 17 of the 598 shared OTUs were significantly different between NWP1 and NWP2 juveniles (Fig. 5B).

### Cryptic species-specific microbiome

In addition to the core microbiome at the species complex level, we clarified unique shared OTUs in specific cryptic species. Fourteen OTUs were only observed in NWP1, while six were only found to be shared between adults and juveniles of NWP2. We ran sequences of unique and shared OTUs in two cryptic species through NCBI BLAST, which identified species names with NCBI IDs. Next, we generated phylograms between identified OTUs using Maximum Likelihood analysis based on the Kimura 2-parameter model (Supplementary Fig. 4). Interestingly, even though its gut was dominated by *Actinobacteria* in the adult stage, four of the six unique OTUs in NWP2 were *Proteobacteria* (i.e. *Neisseria* sp., *Aquabacterium* sp., *Rheinheimera* sp.and *Chujaibacter* sp.). At the same time, three *Actinobacteria* species (*Corynebacterium* sp., *Agrococcus* sp. and *Janibacter* sp.) were only detected in NWP1. Moreover, we detected two closely related *Neisseria* spp. in the gut microbiota of these two different cryptic species.

### Potential impact of seawater microflora on the fish gut microbial community

The influence of host genetics on the gut microbiota was partly reflected in the similarities in communities among juvenile fishes of two closely-related cryptic species, as well as the observation that compositions harboured almost identical membership in all the fish gut samples. However, the clear distinction between structures of gut microbial compositions in adults belonging to three different cryptic species of grey mullet, suggests that exogenous factors may also influences the grey mullet gut microbiota. To test whether migration history is associated with differences in the structure of grey mullet gut microbial composition, we compared our fish gut microbiota with historical seawater microflora of 33 seawater samples from previous studies (Supplementary Table 3)^18–21^. The historical seawater data were collected in the East and South China Seas along the migratory tracts of three cryptic species in the grey mullet species complex suggested by Shen *et al*.^15^.

**Figure 6:**
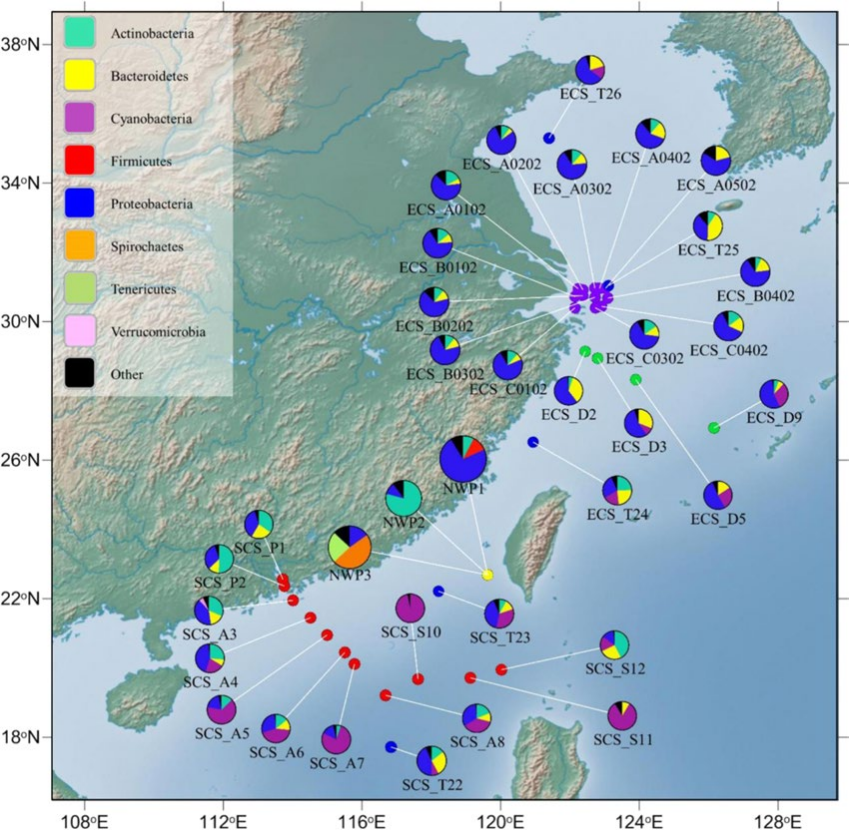
Map of collected fish gut microbiota and historical seawater microflora. The pie charts show the relative abundance of microbiota in collected adult grey mullet gut samples (yellow point) and seawater samples including 33 seawater samples published from four previous studies: Dong *et al*., 2013 (green points); Zhang *et al*., 2014 (red points); Zheng *et al*., 2016 (blue points), and Wu *et al*., 2017 (purple points). The colours in the pie chart present the relative abundance of different bacterial phyla, listed in top-left box, in each individual seawater sample or the grey mullet cryptic species. Name of fish groups and seawater samples are under their respective pie chart. NWP1, NWP2, and NWP3 indicate adults of three cryptic species of grey mullet *M. cephalus* in the Taiwan Strait. The map was created in GenGIS software^58^. Detailed information on seawater samples are provided in Supplementary Table 5.

Our analysis at the phylum level found that the gut microbial composition of NWP1 and seawater-microbiota in the ECS had similar compositions; in particular, most were dominated by *Proteobacteria*, but not *Actinobacteria* or *Firmicutes* (Fig. 6). A dendrogram was used to show the Pearson correlation coefficient results, which were calculated to elucidate the correlation between the fish microbiota and seawater microbes. Clear clusters were identified between mullet gut microbial communities of AD_NWP1 samples and many sea water microbial compositions in ECS. In contrast, the gut microbiota of fish belonging to cryptic species NWP2 were correlated vigorously with seawater samples SCS_P1, SCS_P2, SCS_A3, SCS_A4, SCS_S12 and ECS_T24, the bacterial communities of which were all dominated by *Actinobacteria* (Fig. 6, Supplementary Fig. 5). The dendrogram did not show any close clustering between gut microbial compositions in adult NWP3 and historical seawater data.

## Discussion

The gut microbiota has a fundamental influence on host fitness by contributing to host metabolic capabilities, immunity levels and development. The gut microbial community is, in turn, influenced by several factors, such as genetics, developmental stages, environment and food intake^1–3^. Migratory animals experience extreme conditions during transit that may strongly impact gut microbiota, but this remains poorly understood. Here, we characterize the structure and membership of gut microbial communities in three cryptic species (NWP1, NWP2, and NWP3), of the migratory fish species complex *Mugil cephalus*. Multiple comparisons among adult and juvenile groups of different cryptic species were conducted and showed that, while distinct bacterial communities were observed in the adult fish of different cryptic species, there was still a core microbiome in all fish samples, which was dominated by *Proteobacteria, Firmicutes*, and *Actinobacteria*. This demonstrates that, despite the dynamics of bacterial relative abundance, the gut microbial communities of *M. cephalus* maintain an inflexible proportion of specific bacteria groups inside their gastrointestinal tracts. This finding is consistent with observations in humans^4^ and other animals such as crickets^22^. We suggest that the similarities in membership, not the structure, of the gut microbiota in different cryptic species of *Mugil cephalus* might be highly correlated with their close genetic backgrounds. This supports the hypothesis that fish hosts selectively filter ideal/beneficial microbes from specific exogenous species pools to shape their own gut microbiota so as to keep them healthy and increase their survival ability^23^.

At the family level, the gut microbiota in NWP1 and NPW2 samples were dominated by *Moraxellaceae* (Phylum *Proteobacteria*) and a member of a family in order *Actinomycetales* (Phylum *Actinobacteria*), respectively (Supplementary Fig. 3). *Moraxellaceae* and *Actinomycetales* are popular bacteria groups found in the gut microbiota of many fish species based on analyses of high throughput sequencing data^24–27^. Surprisingly, this study found that *Brevinemataceae* (phylum *Spirochaetes*) and *Mycoplasmataceae* (phylum *Tenericutes*) were the most abundant bacterial families in the gut microbiota of NWP3 adults. A diverse range of *Spirochaetes* are regularly found in marine sediment, deep within soil and in the digestive tracts of arthropods and several mammals. *Spirochaetes* is commonly detected at low abundances in fish gut microbial communities; however, Givens^28^ reported that one of the three barracuda examined contained a community comprised of 99% *Spirochaetes* and the three *Mahi mahi* (common dolphinfish) studied contained 64–98% *Spirochaetes*. We believe that the gut microbiota of marine fish is extremely diverse due to complex influence from various genetic factors and differences in gut habitats. *Clostridiaceae, Streptococcaceae* (phylum *Firmicutes*) and *Vibrionaceae* (phylum *Proteobacteria*) were represented in the juvenile grey mullets’ gut microbial communities. *Vibrionaceae*, representing *Vibrio* bacteria, has been observed as a pathogen group in many animals^29–31^. *Vibrionaceae*, represented by the genera *Vibrio* and *Photobacterium* in the mullet intestinal tract, is one of the most important bacteria families in aquaculture, and both its pathogenic and probiotic species are commonly associated with marine animals^32^.

Our comparison also showed that there is significantly higher diversity in juvenile samples than adult ones. This finding contrasts with observations of the gut microbiota in humans and other animals^33^. However, our results are consistent with the conclusion presented by Stephens *et al*.^34^, who suggested that the gut microbiota in zebrafish had significantly lower richness and diversity in adults than younger fish. In addition, it was reported that juveniles of wild Atlantic salmon, *Salmo salar*, contained a more diverse gut microbiota than those in older life stages^35^. The diversity of food sources of juvenile fish might be one of the reasons for the similarities observed across fish gut microbiota. Juvenile grey mullets (*M. cephalus*) are believed to use several food sources in natural environments for a range of organisms, from copepods and small zooplankton to detritus particles and microalgae^36–38^. We observed high abundances of *Cyanobacteria—*represented by the family *Synechococcaceae* and genus *Synechococcus—*in juvenile gut microbiota, especially in NWP1 juveniles. *Synechococcus* is distributed widely in ocean ecosystems as one of the most common picoplanktonic marine *Cyanobacteria* genera^39^. Our findings suggest that the presence of these bacteria in grey mullet juveniles may be part of not only the fish gut microbiota, but also bacterioplankton and be ingested along with fish hosts’ food items.

Our finding that gut microbial communities in adult fishes of different cryptic species diverged supports our hypothesis about the impact of migration history on the gut microbiota. Therefore, to test whether seawater microflora along the suggested migratory tracts of the three cryptic species are associated with fish gut microbiota, we compared our fish gut data with historical seawater microbial communities^18–21^. It is believed that microorganisms in seawater change over different timescales according to various biological and non-biological forces in the surrounding environment^40^. However, seawater microbial community data from long-term time series in several previous projects from (sub)tropical to polar regions (BATS (Bermuda Atlantic time-series study)^41,42^, HOT (Hawaiian Ocean Time-series)^43^, LMO (Linnaeus Microbial Observatory)^44^, and SPOT (San Pedro Ocean Time-series)^45,46^) suggested that seasonal variation in ocean microbial communities is weakest in sub-tropical regions^40^. Our target species, the grey mullet (*Mugil cephalu*s), is mostly distributed in tropical and subtropical zones (between latitudes 42°N and 42°S)^47^, and all fish samples in this study were observed in the Taiwan Strait (22.73°N, 119.60°E). In addition, it was also suggested that there are “internal feedback mechanisms” inside the microbial community—including mutualism, competition, parasitism and commensalism—that can maintain community’s stability over several years^40^. Therefore, although our fish gut microbiota were observed at different times with historical marine water microflora, the association between our grey mullet gut microbial community results and published seawater microbial compositions is reliable. Nevertheless, our analysis was only at the phylum level. Our comparison showed that gut microbial communities in AD_NWP1 were highly correlated with seawater samples from the ECS, whereas compositions in AD_NWP2 were highly correlated with some specific seawater samples near the shore in the SCS, a proposed *M. cephalus* nursing area. This finding contrasts with results suggested by previous studies that fish gut microbial compositions are rarely influenced by environmental conditions such as water, especially in the mature/adult stage^23,34^. Starvation and osmoregulation in migratory fish could explain this observation. It was proposed that starvation is an opportunity for some specific bacterial groups to increase their competitive ability by utilizing alternative energy sources and then enhancing their relative abundances, as demonstrated with the bacteria inside the intestinal tract of the Asian seabass^48^.

In conclusion, the gut microbiota in different cryptic species of the species complex *Mugil cephalus* are heavily impacted by host genetics, as shown by the presence and high abundance of a core group of microbes (i.e. all sample groups had similar gut microbiota compositions), but strongly structured through life history such as long-distance migration (i.e. distinct gut microbial community structures in the adult stage of different migratory cryptic species). Although the fish sample size is quite small and samples were not taken at the same time as ocean samples were, the observed association between fish gut microbial community and seawater microflora along its migration tracts provides new insights into the host-gut microbiota interaction together with the potential interaction between aquatic animals and their environment in extreme conditions or during perturbations. Further studies with a broader interspecific comparison are highly recommended to investigate in more detail the effects of this exogenous factor on shaping the structure of gut microbiota in aquatic animals. Lastly, clarification of specific invasion microbes and their functions inside the gut microbial community is needed to determine how gut microbiota support their fish host during long-distance migration.

## Materials and Methods

### Sample collection

Fertile female (i.e. fish with fully ripe internal egg masses in their ovaries) migratory grey mullets, estimated to be 3–4 years old fish, were collected in Kaohsiung offshore during their spawning season from mid-November 2016 to mid-January 2017. We specifically chose this site to collect specimens of all three grey mullet cryptic species migrating to the Taiwan Strait, following the migratory tracts suggested by Shen *et al*.^15^. In addition, mullet juveniles were collected from the Danshui estuary in February 2017 (n = 12). Collected fish samples were immediately preserved on dry ice and stored at −20 °C. In the laboratory, the fish surface was sterilized with 70% ethanol and sterile water, then dissected under a hood. The gut contents were kept in 2 mL Eppendorf tubes and preserved at −80 °C until DNA extraction. For adult samples, the gut contents of the last 10 cm of the hindgut were collected for further analysis. For juvenile samples, the whole intestine was used, including both intestinal contents and epithelial associated microbes. Standard length and body weight were also recorded. We could not detect sex by visualization in young grey mullets. All animal procedures were approved by the Academia Sinica Institutional Animal Care and Utilization Committee (AS IACUC) (approved protocol #13-09-576). All experimental methods were performed in accordance with the relevant approved guidelines and regulations.

### DNA extraction and 16S rRNA amplicon sequencing

DNA was extracted from the gut contents of adult and juvenile mullet samples using the DNeasy Blood & Tissue Kit (Qiagen, Venlo, Netherlands) according to the manufacturer’s protocol. DNA concentration was measured with a NanoDrop ND-1000 spectrophotometer (NanoDrop. Technologies, Wilmington, DE). A universal eubacterial primer pair including the forward primer 27F (5′-AGAGTTTGATCMTGGCTCAG-3′) and the reverse primer 335R (5′-GCTGCCTCCCGAGGAGT-3′)^6,49^ combined with an adapter sequence and sample barcodes were used to target specific 16S rRNA gene regions (V1 and V2) by PCR amplification. Extracted DNA samples were diluted to 20 µg/mL directly before PCR processing. The total volume of the PCR mixture was 40 µL, and contained 24 µL 2X Phusion Flash High-Fidelity PCR Master Mix (Finnzymes Oy, Finland), 4 µL of each primer (2 µM), 4 µL template DNA and 4 µL nuclease-free water. The cycling conditions consisted of an initial denaturation at 95 °C for 5 min; followed by 30 cycles of denaturation at 95 °C for 1 min, annealing at 55 °C for 30 s, extension at 72 °C for 1 min; and a final extension at 72 °C for 10 min. The purified amplified PCR products were observed by gel electrophoresis before being extracted with a NucleoSpin Gel and PCR Clean-up Kit (Machrey-Nagel GmbH & Co) and quantified using the Nanodrop-1000 Spectrophotometer (Thermo Scientific, Wilmington, DE, USA). Equal amounts of PCR products were pooled together following the Illumina standard protocol for 16S rRNA sequencing library preparation. Sequencing was performed using the Illumina MiSeq platform (Illumina, San Diego, CA, USA) with the reagent kit v3 at the NGS High Throughput Genomics Core Facility at Academia Sinica. Because collected samples were randomised and high quality samples were refined, we observed and analysed NGS data from a total 16 adult samples (NWP1 n = 8; NWP2 n = 4; NWP3 n = 4) and 12 juvenile samples (NWP1 n = 6; NWP2 n = 6). Detailed information on all samples in this study is provided in Supplementary Table 1.

### Cryptic species identification

We used the methods described by Shen and his research group^15,16^ to identify cryptic species among the observed samples. In general, we extracted DNA from fish tissue using the DNeasy Blood & Tissue Kit (Qiagen, Venlo, Netherlands) according to the manufacturer’s protocol and conducted a multiplex COI haplotype-specific PCR (MHS-PCR). Cryptic species were detected based on the length of PCR products run on gel agarose (2%). Six PCR product samples were sequenced using Sanger sequencing after being cloned into plasmid vectors. Nucleotide BLAST on Genbank was used to identify sequences. The cloned genes showed more than 97% identity with the COI gene of grey mullet. In addition, a phylogeny tree (not shown) was constructed, and cryptic species samples identified in our study were clustered with corresponding cryptic species from previous studies. As we used the method from previous studies^15,16^, we decided to keep the same abbreviations for the three grey mullet cryptic species(i.e. NWP1, NWP2, and NWP3). The list of fish samples is presented in Supplementary Table 1 and is made up of 16 adult fish samples—eight NWP1, four NWP2, and four NWP3—and 12 juvenile fish samples—six each of NWP1 and NWP2. We did not observe any juvenile NWP3 samples, probably because NWP3 juveniles are scarce in Taiwanese waters (unpublished data). However, comprehensive data on juvenile and adult samples of NWP1 and NWP2 suggested that more information is needed on the differences in gut microbial diversity among the different life stages, as well as the potential influence of migratory tracts on the intestinal microbiota.

### Historical ocean water microflora data

The three cryptic species used in this study are believed to migrate from the East and South China Seas to the Taiwan Strait during their spawning season^15^. In particular, fertile NWP1 were proposed to migrate to the Taiwan Strait following the China coastal current, while NWP2 and NWP3 individuals reach their spawning area by following the Kuroshio Current. Therefore, to investigate the potential impacts of migration on the structure of gastrointestinal microbiota in migratory grey mullets, historical seawater microbial community data on grey mullet migration routes were adapted from four previous studies^18–21^. The list of cited data and detailed information on the samples are listed in Supplementary Table 4. We specifically chose data from those studies because their methods were similar, using 16S rRNA amplicon sequencing to clarify the microbial community structure from environmental samples. Raw 16S rRNA sequences from seawater samples were combined with our raw fish gut microbiota data and analysed together following the sequence analysis process.

### Taxonomic assignment and statistical analysis

All raw paired-end Illumina amplicon sequences in FASTQ format were processed with MOTHUR v.1.36.1^50^. During pre-processing, raw paired-end sequences (including forward and reverse sequences) were merged using the fastq_mergepairs command in USEARCH. Next, the sequences were quality-trimmed using command trim.seqs in Mothur (www.mothur.org/wiki/Trim.seqs) with a quality score of 30, number of errors in the barcode (maximum of 1), number of errors in the primer (maximum of 2), and length from 250 to 500 bp^51^. After picking a reference sequence using the command unique.seqs in Mothur, the partial 16S rRNA sequences were hierarchically clustered at 97% using command cluster_otus in USEARCH, which also removes chimeras from all libraries. Final OTUs were taxonomically classified using the classify.seqs script in Mothur against GreenGenes 13_8 database as a reference^52^. The final step was to remove OTUs derived from the Chloroplast, Mitochondria, Eukaryota, or unknown kingdoms and to generate an OTU table.

Measurements of alpha diversity—including observed number of OTUs, richness (Chao), and community diversity (Shannon and inverse Simpson’s index)—were calculated using the collect.single script in Mothur. The permutation T-test in R using the RVAideMemoire package^53^ tested for differences in alpha diversity between adults of different cryptic species as well as between adults and juveniles of the same cryptic species. A *p* value < 0.05 was considered statistically significant.

Microbiota β-diversity was studied using the Bray-Curtis dissimilarity index and weighted UniFrac distance. Later, β-diversities of grey mullet gut microbial communities were visualized by generating non-metric multidimensional scaling (NMDS) plots in R using the vegan package^54^. A permutational multivariate analysis of variance (PERMANOVA) test was conducted using the pairwiseAdonis package^55^ to compare microbial community structures among the cryptic species as well as inter-specific cryptic species at different life stages.

Next, the phylogenetic tree of shared OTUs within cryptic species was constructed using Maximum Likelihood analysis based on the Kimura 2-parameter model in MEGA 7^56^. Box plots, bar charts and heatmaps were created using R and STAMP^57^. Geospatial biodiversity mapping analysis of fish gut microbial compositions and historical seawater microflora was performed using GenGIS II^58^. This method was used to determine the potential association between microbiota in historical seawater along migratory routes and specific gut microbial communities in grey mullets. Finally, a dendrogram based on the Pearson correlation coefficient was generated using Generalized Association Plots (GAP v0.2.7)^59^.

### Data deposition

Raw sequences from 16S gene profiling were deposited into the National Center for Biotechnology Information (NCBI) under project accession number PRJNA494515 through the following SRA study accession numbers: SRR7958800-SRR7958827

## Supplementary information

Supplementary information.
