# Supplementary material for: Structure and membership of gut microbial communities in multiple fish cryptic species under potential migratory effects
Source: Sci Rep. 2020 May 5;10:7547. doi: 10.1038/s41598-020-64570-8 (PMC7200715; doi:10.1038/s41598-020-64570-8)
Supplement: Supplementary file 1 — Supplementary information. [file 41598_2020_64570_MOESM1_ESM.pdf]

Structure and membership of gut microbial communities in multiple fish cryptic species under potential migratory effects

My Hanh Le[1,2,3,4] **and Daryi Wang**[1*]

[1] Biodiversity Research Center, Academia Sinica, Taipei, Taiwan

[2] Department of Life Science, National Taiwan Normal University, Taipei, Taiwan

[3] Biodiversity Program, Taiwan International Graduate Program, Academia Sinica and National Taiwan Normal University, Taipei, Taiwan

[4] Institute of Ecology and Biological Resources, Viet Nam Academy of Science and Technology, Hanoi, Viet Nam

***Correspondence:** Dr. Daryi Wang dywang@gate.sinica.edu.tw

Supplementary Information

Supplementary Table 1. List of grey mullet samples and 16S rRNA gene sequences following sequence filtering pipelines

ID	Sampling region	Collecting date	Gender	Weight (gram)	Length (cm)	Raw	Merged	Trimmed	Classified	Percentage	No. of OTUs
JV1_NWP1	Taiwan: Tamshui estuary	2017-02-23	NA	0.23	2.644	301162	222704	197730	84207	27.96	640
JV2_NWP1	Taiwan: Tamshui estuary	2017-02-23	NA	0.23	2.482	186477	137578	123264	111470	59.78	578
JV3_NWP1	Taiwan: Tamshui estuary	2017-02-23	NA	0.2	2.657	258027	191568	170669	76317	29.58	602
JV4_NWP1	Taiwan: Tamshui estuary	2017-02-23	NA	0.21	2.471	130218	96725	86572	75613	58.07	382
JV5_NWP1	Taiwan: Tamshui estuary	2017-02-23	NA	0.19	2.377	279715	211093	188669	78108	27.92	636
JV6_NWP1	Taiwan: Tamshui estuary	2017-02-23	NA	0.16	2.689	231673	175112	157537	148400	64.06	559
JV1_NWP2	Taiwan: Tamshui estuary	2017-02-23	NA	0.16	2.627	148555	110957	99361	92743	62.43	437
JV2_NWP2	Taiwan: Tamshui estuary	2017-02-23	NA	0.21	2.507	149329	110101	98868	89676	60.05	563
JV3_NWP2	Taiwan: Tamshui estuary	2017-02-23	NA	0.29	2.862	181451	132781	117926	115862	63.85	255
JV4_NWP2	Taiwan: Tamshui estuary	2017-02-23	NA	0.28	2.844	133219	97014	86796	79961	60.02	566
JV5_NWP2	Taiwan: Tamshui estuary	2017-02-23	NA	0.25	2.572	126998	96182	85812	78507	61.82	560
JV6_NWP2	Taiwan: Tamshui estuary	2017-02-23	NA	0.16	2.268	144178	104450	92988	88140	61.13	550
AD1_NWP1	Taiwan: Kaohsiung offshore	2017-01-11	F	NA	40.191	215013	170949	156081	151885	70.64	119
AD2_NWP1	Taiwan: Kaohsiung offshore	2017-01-11	F	NA	40.728	216088	173494	158984	153171	70.88	133
AD3_NWP1	Taiwan: Kaohsiung offshore	2017-01-11	F	NA	37.609	281062	221159	202250	198780	70.72	134
AD4_NWP1	Taiwan: Kaohsiung offshore	2017-01-11	F	NA	40.611	265042	211031	193370	187606	70.78	125
AD5_NWP1	Taiwan: Kaohsiung offshore	2017-01-11	F	NA	41.158	279914	224150	205126	200144	71.50	117
AD6_NWP1	Taiwan: Kaohsiung offshore	2017-01-11	F	NA	31.628	247614	195467	177122	172823	69.80	111
AD7_NWP1	Taiwan: Kaohsiung offshore	2017-01-11	F	NA	38.004	275752	219133	201277	190982	69.26	136
AD8_NWP1	Taiwan: Kaohsiung offshore	2017-01-11	F	NA	44.295	209452	167420	152632	149554	71.40	110
AD1_NWP2	Taiwan: Kaohsiung offshore	2016-12-10	F	NA	44.075	238420	186110	166273	157571	66.09	163
AD2_NWP2	Taiwan: Kaohsiung offshore	2016-12-10	F	NA	39.995	223163	174107	158058	151981	68.10	142
AD3_NWP2	Taiwan: Kaohsiung offshore	2016-12-10	F	NA	39.489	201463	155191	140797	128128	63.60	254
AD4_NWP2	Taiwan: Kaohsiung offshore	2016-12-10	F	NA	38.785	195066	151121	138975	119844	61.44	149

AD1_NWP3	Taiwan: Kaohsiung offshore	2016-12-10	F	NA	41.165	323649	203946	180770	176733	54.61	107
AD2_NWP3	Taiwan: Kaohsiung offshore	2016-12-10	F	NA	47.983	305661	189300	168225	165712	54.21	112
AD3_NWP3	Taiwan: Kaohsiung offshore	2016-12-10	F	NA	40.884	253450	158301	139883	136029	53.67	192
AD4_NWP3	Taiwan: Kaohsiung offshore	2016-12-10	F	NA	43.826	292757	176584	155740	144904	49.50	255

Supplementary Table 2. Permutation T-test results of alpha diversity in the grey mullet gut microbiota

Groups	Sob		Chao		Shannon		Inverse_Simpson	
	MD	*p*	MD	*p*	MD	*p*	MD	*p*
**AD1 – AD2**	53.9	**0.006**	50.43	**0.028**	1.14	**0.008**	3.22	**0.002**
**AD1 – AD3**	43.4	0.176	46.37	0.098	0.36	0.338	0.61	0.75
**AD2 – AD3**	10.5	0.808	4.06	0.946	0.78	0.216	3.83	**0.04**
**AD1 – JV1**	443.1	**0.004**	450.06	**0.002**	1.51	**0.002**	16.4	**0.006**
**AD2 – JV2**	311.5	**0.014**	326.9	**0.02**	1.68	0.078	9.09	**0.038**
**JV1 – JV2**	77.7	0.256	72.73	0.238	0.97	0.142	10.53	0.096

AD1, NWP1 adult; AD2, NWP2 adult; AD3, NWP3 adult; JV1, NWP1 juvenile; JV2, NWP2 juvenile. NWP1, NWP2, and NWP3 are the three cryptic species of grey mullet *Mugil cephalus* in the Taiwan Strait.
MD indicates mean difference between two groups.
Statistically significant values are presented in bold.

Supplementary Table 3. PERMANOVA results based on Bray-Curtis and weighted UniFrac distance matrices using abundance data of gut bacterial community in grey mullets at the genus level (shown in Figure 4)

Pairs	Total Df	Bray Curtis		Weighted UniFrac	
		R^2	Adjusted *p*	R^2	Adjusted *p*
**AD1 - AD2**	11	0.80	0.0067 **	0.87	0.008 **
**AD1 - AD3**	11	0.74	0.007 **	0.76	0.0067 **
**AD2 - AD3**	7	0.80	0.038 *	0.82	0.033 *
**AD1 - JV1**	13	0.73	0.0067 **	0.67	0.005 **
**AD2 - JV2**	9	0.56	0.0067 **	0.49	0.0087 **
**JV1 - JV2**	11	0.16	0.061	0.16	0.214

AD1, NWP1 adult; AD2, NWP2 adult; AD3, NWP3 adult; JV1, NWP1 juvenile; JV2, NWP2 juvenile. NWP1, NWP2, and NWP3 are the three cryptic species of grey mullet *M. cephalus* in the Taiwan Strait.
Df: Degrees of freedom
p values based on 999 permutations, adjusted by Benjamini-Hochberg method
*$p<0.05$ **$p<0.01$

Supplementary Table 4. Number of shared OTUs and their relative abundance between gut microbial communities in different cryptic species at different life stages

Group	No. of shared OTUs	Relative abundance of shared OTUs	Shared OTUs between all samples
AD1 – AD2	157	99.19% – 97.8%	122
AD1 – AD3	168	98.75% – 91.75%	
AD2 – AD3	194	98.87% – 90.58%	
AD1 – JV1	198	99.55% – 56.04%	
AD2 – JV2	264	99.67% – 79.1%	
JV1 – JV2	598	99.75% – 99.87%	

AD1, NWP1 adult; AD2, NWP2 adult; AD3, NWP3 adult; JV1, NWP1 juvenile; JV2, NWP2 juvenile. NWP1, NWP2 and NWP3 are the three cryptic species of grey mullet *M. cephalus* in the Taiwan Strait.
Shaded relative abundance of shared OTUs correspond to shaded groups in the first column.
OTUs were liberally classified at 97% identity at the genus level.

Supplementary Table 5. List of historical seawater samples used in this study

Site Id	Date	Latitude	Longitude	Distance (miles)[a]	Sample Depth (m)	Habitat Type	Geographic Location	Cited from
ECS_D2	May, 2011	29.1758	122.4114	478	1	ocean	East China Sea	Dong et al., 2013
ECS_D3	May, 2011	28.9773	122.7677	474	1	ocean	East China Sea	Dong et al., 2013
ECS_D5	May, 2011	28.3628	123.8683	506	1	ocean	East China Sea	Dong et al., 2013
ECS_D9	May, 2011	26.9689	126.1191	502	1	ocean	East China Sea	Dong et al., 2013
SCS_P1	Jan, 2010	22.6063	113.705	376	0.5	estuary	South China Sea	Zhang et al., 2014
SCS_P2	Jan, 2010	22.4092	113.7516	374	0.5	estuary	South China Sea	Zhang et al., 2014
SCS_A3	Jan, 2010	21.99818	113.9988	361	5	ocean	South China Sea	Zhang et al., 2014
SCS_A4	Jan, 2010	21.49672	114.5015	337	5	ocean	South China Sea	Zhang et al., 2014
SCS_A5	Jan, 2010	20.99705	114.9789	320	5	ocean	South China Sea	Zhang et al., 2014
SCS_A6	Jan, 2010	20.49689	115.4891	306	5	ocean	South China Sea	Zhang et al., 2014
SCS_A7	Jan, 2010	20.1623	115.7672	304	5	ocean	South China Sea	Zhang et al., 2014
SCS_A8	Jan, 2010	19.26202	116.6623	306	5	ocean	South China Sea	Zhang et al., 2014
SCS_S10	Jan, 2010	19.72681	117.5972	244	5	ocean	South China Sea	Zhang et al., 2014
SCS_S11	Jan, 2010	19.76478	119.1056	207	5	ocean	South China Sea	Zhang et al., 2014
SCS_S12	Jan, 2010	19.99886	119.9997	190	5	ocean	South China Sea	Zhang et al., 2014
SCS_T22	May, 2014	17.77	116.82	387	4	ocean	South China Sea	Zheng et al., 2016
SCS_T23	May, 2014	22.26	118.19	96	4	ocean	South China Sea	Zheng et al., 2016
ECS_T24	May, 2014	26.56	120.91	277	4	ocean	East China Sea	Zheng et al., 2016
ECS_T25	May, 2014	31.07	123.08	614	4	ocean	East China Sea	Zheng et al., 2016
ECS_T26	May, 2014	35.32	121.37	876	4	ocean	East China Sea	Zheng et al., 2016
ECS_A0102	July, 2015	30.98	122.2117	592	2	estuary	East China Sea	Wu et al., 2017
ECS_A0202	July, 2015	30.94667	122.345	592	2	estuary	East China Sea	Wu et al., 2017
ECS_A0302	July, 2015	30.99833	122.6883	601	2	estuary	East China Sea	Wu et al., 2017
ECS_A0402	July, 2015	30.99167	122.825	604	2	estuary	East China Sea	Wu et al., 2017
ECS_A0502	July, 2015	30.995	122.9983	608	2	estuary	East China Sea	Wu et al., 2017
ECS_B0102	July, 2015	30.75667	122.1167	575	2	estuary	East China Sea	Wu et al., 2017
ECS_B0202	July, 2015	30.78167	122.3417	581	2	estuary	East China Sea	Wu et al., 2017
ECS_B0302	July, 2015	30.735	122.6967	584	2	estuary	East China Sea	Wu et al., 2017
ECS_B0402	July, 2015	30.71167	123.0633	591	2	estuary	East China Sea	Wu et al., 2017
ECS_C0102	July, 2015	30.43167	122.115	554	2	estuary	East China Sea	Wu et al., 2017
ECS_C0302	July, 2015	30.43	122.705	565	2	estuary	East China Sea	Wu et al., 2017
ECS_C0402	July, 2015	30.47167	122.8833	571	2	estuary	East China Sea	Wu et al., 2017

[a]: The column shows data of estimated distance from the collected seawater point (previous study) to the collected fish point (present study). The formula used to calculate distance does not take into account the non-spheroidal (ellipsoidal) shape of the Earth.

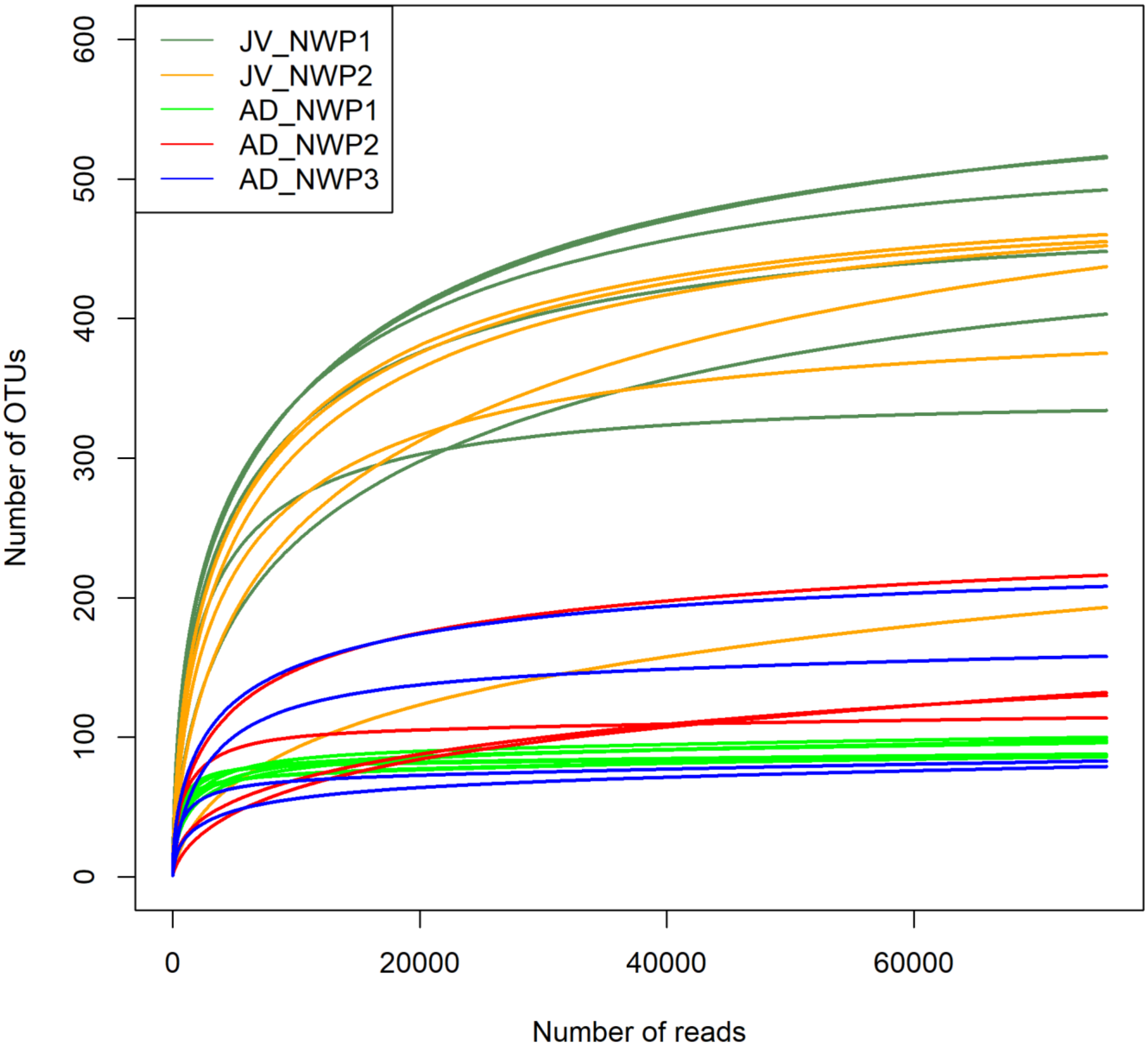

Supplementary Figure 1. Rarefaction analyses of the observed number of OTUs in the grey mullet gut microbiome. Individual rarefaction curves for observed grey mullet samples in this study. Light green curves for NWP1 adult samples, red curves for communities in NWP2 adults, blue curves for NWP3 adults, and dark-green and yellow curves for juvenile samples of NWP1 and NWP2, respectively. NWP1, NWP2 and NWP3 are the three cryptic species of grey mullet *M. cephalus* in the Taiwan Strait.

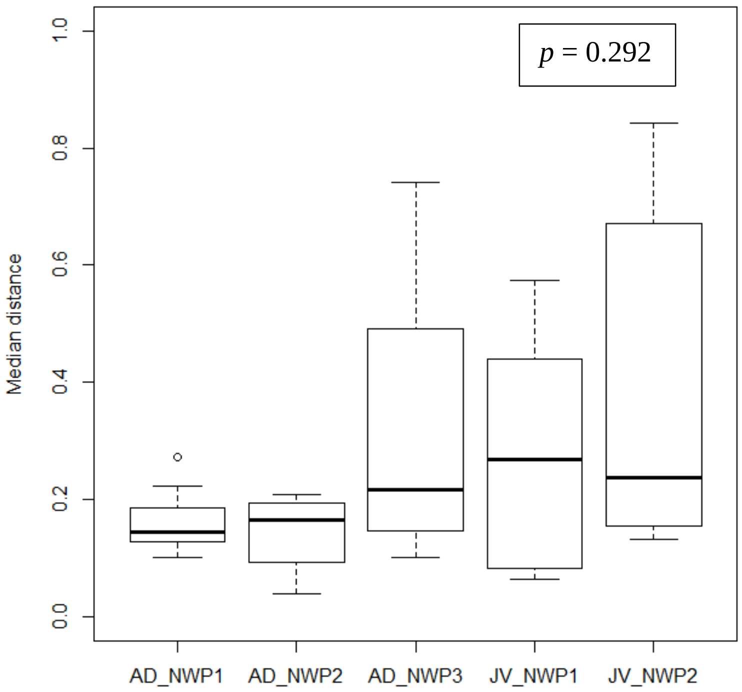

Supplementary Figure 2. Boxplot for inter-specific variation within grey mullet groups. Black line: median; lower and upper boxes: 25 and 75 percent quartiles, respectively. X axis shows the group IDs: JV: juvenile, AD: adult. NWP1, NWP2, and NWP3 are the three cryptic species of grey mullet *M. cephalus* in the Taiwan Strait.

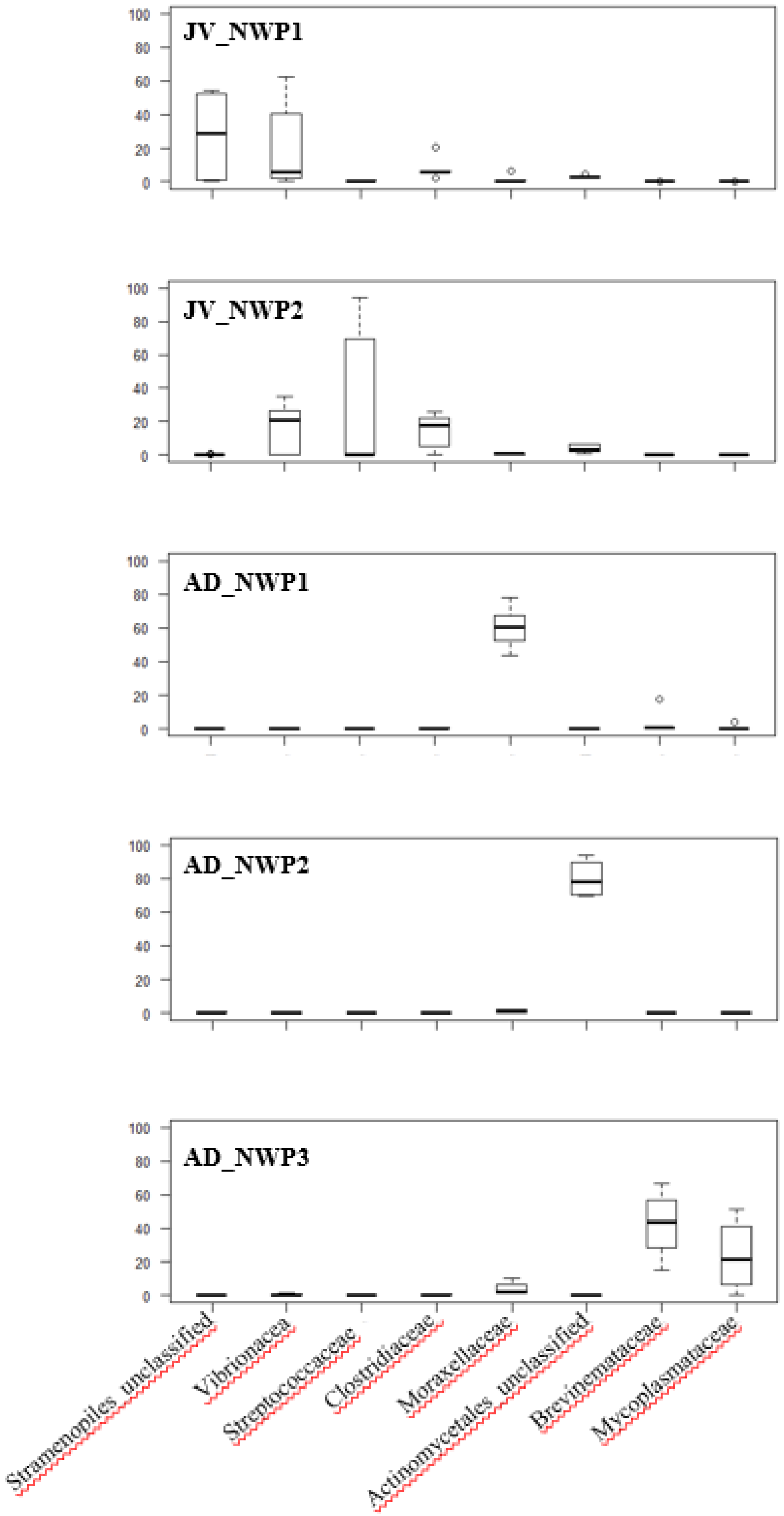

Supplementary Figure 3. **Relative abundance of dominated families in *M. cephalus* gut microbial communities.** JV: juvenile; AD: adult; NWP1, NWP2, and NWP3 are the three cryptic species of grey mullet *M. cephalus* in the Taiwan Strait.

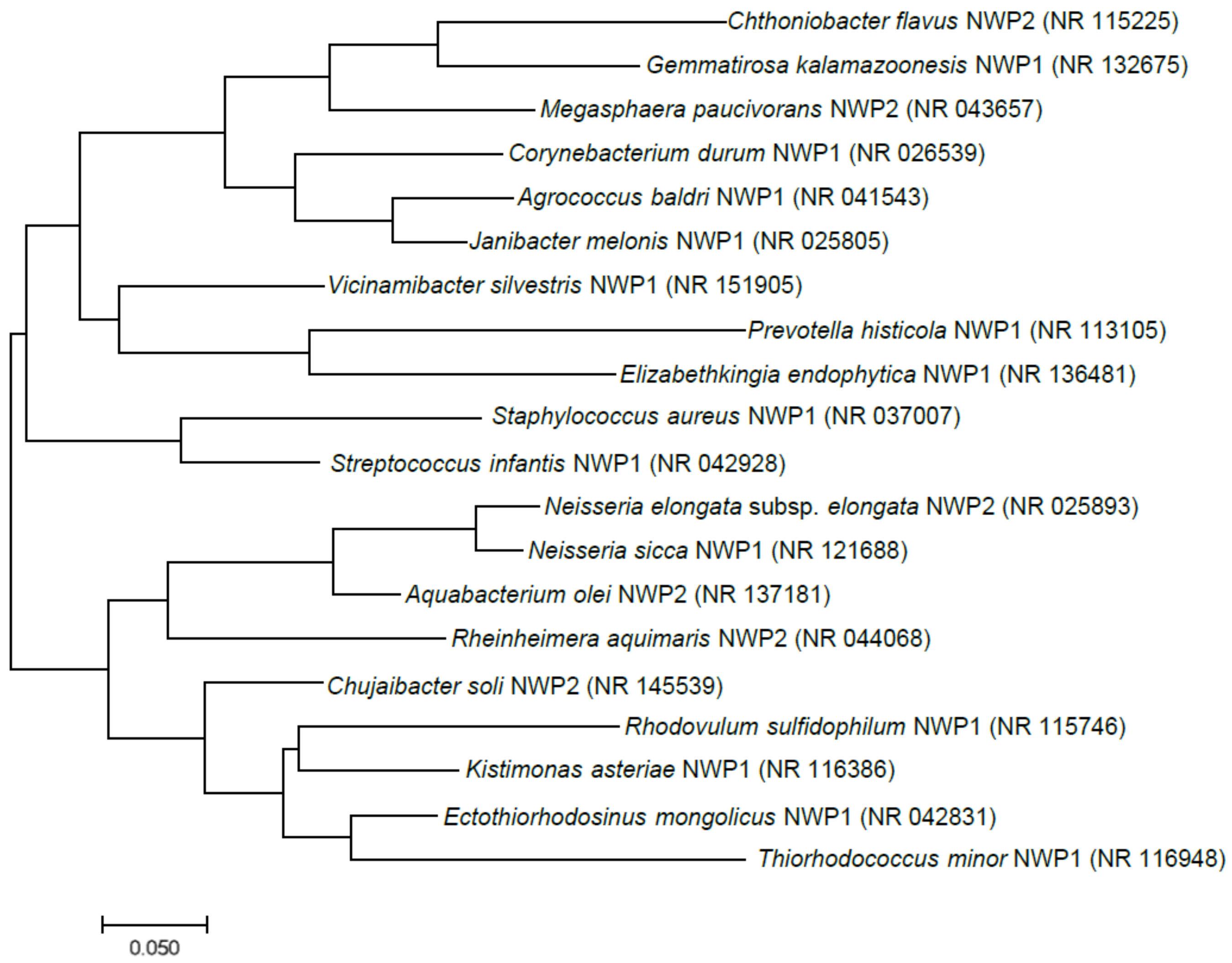

Supplementary Figure 4. The phylogenetic tree of species identified based on cloned amplicons of 16S rRNA genes of unique and shared OTUs between specific grey mullet cryptic species. Species were identified through NCBI BLAST including NCBI ID; NWP1 and NWP2 are two cryptic species of *M. cephalus*. This tree topology is identical to that of the maximum likelihood analysis based on the Kimura 2-parameter model.

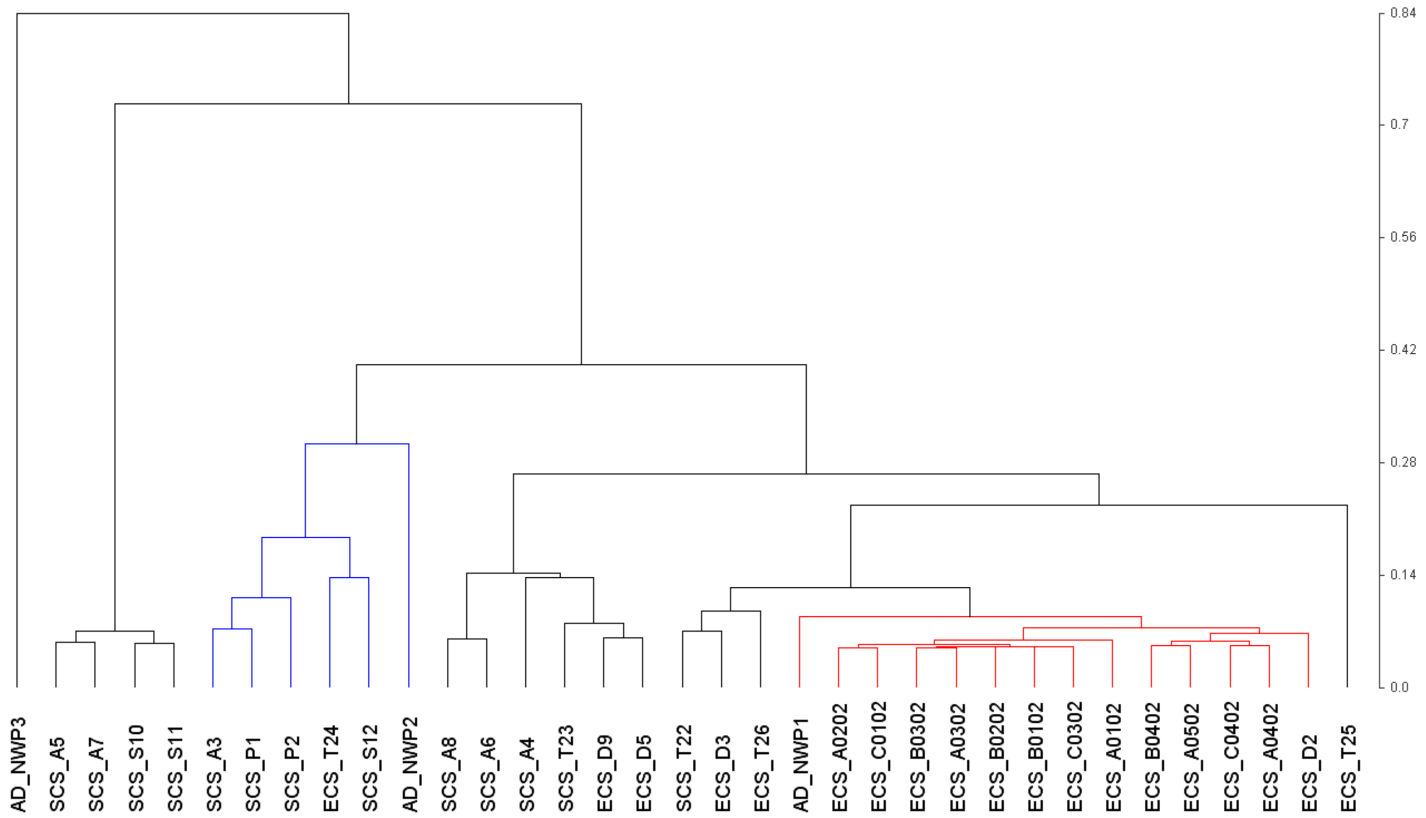

Supplementary Figure 5. Dendrogram cluster analysis (Pearson's correlation coefficients and single linkage) for the gut microbial compositions of grey mullet and seawater microflora generated by GAP v0.2.7. AD_NWP1, AD_NWP2 and AD_NWP3 are adults of the three cryptic species of grey mullet *M. cephalus* in the Taiwan Strait. Detailed information on seawater samples are provided in Supplementary Table 5.
